# Biological Activity of Coumarin Derivatives as Anti-Leishmanial Agents

**DOI:** 10.1371/journal.pone.0164585

**Published:** 2016-10-21

**Authors:** Vineetha Mandlik, Sohan Patil, Ramanamurthy Bopanna, Sudipta Basu, Shailza Singh

**Affiliations:** 1 National Centre for Cell Science, NCCS Complex, SP Pune University Campus, Ganeshkhind, Pune, India; 2 Department of Chemistry, Indian Institute of Science Education and Research (IISER), Pune, Pashan, Pune, India; Jamia Millia Islamia, INDIA

## Abstract

Cutaneous leishmaniasis affects nearly 0.7 to 1.3 million people annually. Treatment of this disease is difficult due to lack of appropriate medication and the growing problem of drug resistance. Natural compounds such as coumarins serve as complementary therapeutic agents in addition to the current treatment modalities. In this study, we have performed an in-silico screening of the coumarin derivatives and their anti-leishmanial properties has been explored both *in-vitro* and *in-vivo*. One of the compounds (compound 2) exhibited leishmanicidal activity and to further study its properties, nanoliposomal formulation of the compound was developed. Treatment of cutaneous lesions in BALB/c mice with compound 2 showed significantly reduced lesion size as compared to the untreated mice (p<0.05) suggesting that compound 2 may possess anti-leishmanial properties.

## Introduction

Leishmaniasis is a neglected tropical disease that is caused by the parasite ‘*Leishmania*’. Nearly 12 million people worldwide are affected by this disease. There are three forms of leishmaniasis, namely cutaneous, mucocutaneous and visceral forms. Out of these, cutaneous leishmaniasis (CL) is more wide spread and has an higher incidence that the visceral form. CL is more prevalent in parts of Latin America, Middle east and Central Asia. Typical symptoms of CL are the formation of an unpleasant looking skin sores and scars. Treatment of CL is usually done by administration of antimony based compounds (sodium stibogluconate and meglumine antimoniate) in the form of intramuscular injections. However studies reveal that antimonials have their own share of toxicity and side effects. [1] Second line chemotherapy therefore is now focusing on oral anti-fungal compounds, liposomal formulations (Amphotericin B) and topical formulations of paromomycin (Leshcutan). Liposomal amphotericin B (Ambiosome) is also being used for treatment of CL incase of drug resitance to the existing antimonials. Amphotericin B has been shown to be effective against all forms of leishmaniasis, however, the liposomal formulation proves to be expensive, has a lower therapeutic index and is difficult to administer.[2] Paromomycin and Pentamidine are some other compounds that show good efficacy to some species but not to all. Moreover, these drugs are usually administered parentally and this limits their use. [3] Miltefosine (hexadecylphosphocholine) is the only oral drug available that has been reported to induce apoptosis in *L*.*major* though its efficacy in CL is still under consideration. [4,5] With the increasing reports of drug resistance to the available chemotherapeutics, it becomes necessary to identify new compounds that could be used in combination therapy or alone to treat cutaneous leishmaniasis. [6]

Several natural compounds such as alkaloids, phenolic compounds, terpenes and saponins have anti-leishmanial properties. [7,8] Recent studies have also found that coumarins possess anti-leishmanial activity. [9] Two new coumarins i.e. 5-methylcoumarins were isolated from the roots of *Vernonia brachycalyx* and their structures were elucidated using MS and NMR spectroscopy. [10] Coumarin (-) mammae A/BB discovered by Brenzan *et al* shows efficacy over *L*.*amazonensis* with an IC_50_ value < 10 μM. [11] More recently compounds like auraptene (LD_50_ = 30 μM), osthole (IC_50_ = 14.95 μg/ml), coumarin- triazolothiadiazine hybrids (IC_50 =_ 0.8 μM), triclosan-coumarin hybrids (EC_50_ = 9.4; 10.2; 13.5 and 27.5 μg/mL), sesquiterpene coumarins (IC_50_ = 11.5 μg/mL) and indoyl coumarin hybrids (IC_50_ = 12.4 μg/mL to 13.4 μg/mL) have been isolated that have been shown to be active against the promastigote form of *Leishmania major*. [12,13, 14, 15, 16, 17] In the present study, we have screened a set of coumarin derivatives. In our computational screening procedure, we have identified a set of five compounds which were screened for their anti-leishmanial properties. For the ease of presentation, these compounds have been designated as **C1-C5**. The compound 3-(1,3-benzodioxol-5-yl)-2-oxo-2H-chromen-6yl acetate **(C2)** showed the highest anti-leishmanial properties both *in-vitro* and *in-vivo*. To futher enhance the solubility of **C2**, nano-liposomal formulation was prepared and the efficacy of the compound **(C2)** was determined.

## Materials and Methods

### *Insilico* screening

Using coumarin as a search molecule, its derivatives were obtained from the zinc database. [18] An intial filtering of the compounds was performed based on the “Lipinski rule of five” and the “Vebers rules”. [19, 20]. Further screening included the estimation of the tanimoto combo score using ROCS (Rapid overlay of chemical structures). Molecules with a tanimoto combo score greator than 0.6 were included in further analysis. [21] A total of 30 compounds had a tanimoto combo score greator than 0.6. The 2D structures of the all the 30 compounds were sketched using Chemsketch. [22] For the QSAR analysis [23], a total of 28 molecules with known antifungal and antiprotozoan activity were obtained using literature search, these were included in the training set during QSAR analysis. The test set included the 30 compounds obtained post screening with ROCS. The pIC_50_ values of the test compounds was predicted and the top five compounds with the highest pIC_50_ value were selected for further experimentation. These five compounds were not commercially available and the synthesis of the compounds was done by Sigma Aldrich, USA, the schema for the synthesis of the main compound (C2) is shown in S1 Fig. The NMR data and IR data for C2 is shown in S2 and S3 Figs respectively. HPLC confirms the C2 to be of 98.5% purity. (Sigma Aldrich,USA)

### *Invitro* screening

#### Parasite culture

*Leishmania major* promastigotes (MHOM/IL/67/JERICHO II) were maintained at 27°C in RPMI-1640 media supplemented with 20% heat inactivated fetal bovine serum (GIBCO BRL, Grand Island, NY) and antibiotics. All experiments were performed with parasite cultures in the logarithmic phase of growth.

### Anti-leishmanial activity assay

The five compounds **(C1-C5)** were dissolved in DMSO to prepare a stock of 10mM. The stock was further diluted in the culture media (RPMI-1640) such that the final concentration of DMSO was restricted to 0.7% (v/v). Around 1X10^6^ *L*.*major* promastigotes were seeded into each well. Serial dilutions of the drugs were made such that the range of concentrations was from 1–1000 μM. The viability of the parasites was assessed using MTT (3-[4, 5-methylthiazol-2-yl]-2, 5-diphenyltetrazolium bromide) method. Briefly 20μl of MTT (5mg/ml) was added to each well including the controls. Plates were incubated in dark for a time period of 4 h at 27°C. After the incubation period, centrifugation was performed at 6010Xg for 10min at room temperature. The media was replaced by 100 μl of DMSO and absorbance was read spectrophotometrically using a 570nm filter (MultiScan FC, Microplate photometer, Thermofisher).

### Mammalian cell cytotoxicity

To evaluate the cytotoxic effect of the compounds, macrophage cell line (J774) was used. Macrophages were cultured in 96 well microtitre plates (1X10^5^cells/well) in DMEM media (GIBCO BRL, Grand Island, NY) containing heat inactivated 10% FBS and 100 μg/ml penicillin-streptomycin. Cells were maintained at 37°C, 5% CO_2_ for a period of 24 hours. As soon as the cell confluency was achieved, medium was replaced with fresh medium containing compounds **(C2 and C3)** at their IC_50_ concentrations, incubated for a period of 48h. After incubation, control and treated cells were washed with PBS, pH 7.2. 180μl of PBS and 20μl of MTT solution (5mg/ml) was added to each well. Cells were incubated for 4 h at 37°C, 5% CO_2._ The formazan crystals were dissolved in 100 μl of DMSO and the absorbance was read spectrophotometrically using a 570nm filter (MultiScan FC, Microplate photometer, Thermofisher). The percentage of viable cells were calculated as % Cell viability = (A_T_-A_B_/A_C_-A_B_) X 100 were A_T_ is the absorbance of treated wells, A_C_ the absorbance of control wells (not treated), and A_B_ the absorbance of blank wells culture medium and DMSO only). The experiment was carried out in triplicates and a set of two independent experiments were performed. A set of three separate experiments were performed for each drug and the cell viability at IC_50_ concentration was determined.

### Development of nanoliposomal formulation of C2

Nanoliposomes of one of the compounds (compound **2)** were prepared using the thin film hydration method. Compound 2 **(C2)**, L*-α-*phosphatidylcholine (PC) (Sigma-Aldrich), 1,2-distearoyl-*sn*-glycero-3-phosphoethanolamine-N-[amino(polythylene glycol)2000] (DSPE-PEG) (Avanti Polar Lipids, Inc.) and cholesterol (Sigma-Aldrich) 1.0: 2.0: 0.1: 0.5 weight ratio were dissolved in 5 mL dichloromethane (DCM) (Sigma-Aldrich). DCM was evaporated using a rotary evaporator to form thin and uniform lipid film. The lipid film was then hydrated with 1.0 mL H_2_O for 1 h at 60°C with intermittent sonication. The resultant solution was passed through the Sephadex^®^ G-25 (Sigma-Aldrich), sequentially extruded through 200 nm Whatmann polycarbonate membrane using Avanti^®^ mini-extruder (Avanti Polar Lipids, Inc.) The nanoliposomes were stored at 4°C until further use.

#### Size and shape of nanoliposomes

The size, shape and morphology of the nanoliposomes were determined by dynamic light scattering (DLS), field-emission scanning electron microscopy (FESEM) and atomic force microscopy (AFM). Samples were prepared for DLS, FESEM and AFM. Stability of the nanoliposomes was checked as per the procedure mentioned in reference [24]

#### Drug loading

A Calibration curve was plotted in the concentration range of 10 to 30 μM by diluting the 1 mM standard stock solution of the **C2** in dimethyl sulfoxide (DMSO). The absorbance was measured at 256 nm against the corresponding solvent blank. The linearity was plotted for absorbance against concentration. For checking drug loading in nanoliposomes, prepared nanoliposomes were dissolved in DMSO in 5%, 10% and 15% dilution. Absorbance was measured at characteristic wave length against the corresponding solvent blank in 200 μL quartz cuvette and from the calibration curve drug loading was measured in triplicates.

#### Drug release profile

Concentrated drug loaded nanoliposomes (200 μl) were suspended in 200 μL of buffer (pH = 7.4), sealed in a dialysis membrane (MWCO = 500 Dalton) and incubated in 15mL PBS buffer at room temperature with gentle shaking. 200 μL portion of the aliquot was collected from the incubation medium at predetermined time intervals and the released drug was quantified by UV-VIS Spectrophotometer (Chemito Spectrascan UV 2600).

### Cytotoxicity profile of the nanoparticles (C2NL)

To evaluate the cytotoxicity of the nanoliposomes, macrophage cell line (J774) was used. Macrophages (1X10^5^ cells/well) were cultured in 96 well microtitre plate as described above. Cells were maintained at 37°C, 5% CO_2_ for a period of 24 h. After 24h, the medium was replaced with fresh medium containing different concentrations of liposomes (0–30μM) containing **C2**. After the incubation period of 48h, MTT assay was performed. Absorbance was read spectrophotometrically using a 570nm filter (MultiScan FC, Microplate photometer, Thermofisher) and the percentage of viable cells were calculated. The experiment was carried out in duplicates and a set of two independent experiments were performed. The cytotoxicity profile was obtained and the graph was plotted using GraphPad Prism 5.

### Evaluation of anti-leishmanial activity of the synthesized nanoparticles

Lograthimic phase *L*.*major* promastigotes (1X10^6^ cells/well) were seeded in 96 well plates. To evaluate the inhibitory potential of the nanoliposomes, dilutions of the nanoliposomes were prepared in the culture media (RPMI-1640). The range of concentrations was from 0–30μM. The plates were incubated for a time period of 48h. Viability of the parasites was assessed using MTT (3-[4, 5-methylthiazol-2-yl]-2, 5-diphenyltetrazolium bromide) method. Briefly 20μl of MTT (5mg/ml) was added to each well including the controls. Plates were incubated in dark for a time period of 4h at 27°C. After the incubation period, centrifugation was performed at 6010Xg for 10min at room temperature. The media was replaced by 100μl of DMSO and absorbance was read spectrophotometrically using a 570nm filter (MultiScan FC, Microplate photometer, Thermofisher).

### Morphological studies

*L*.*major* promastigotes were exposed to the compound 2 containing nanoliposomes **(C2NL)** and the morphological alterations were investigated by optical microscopy. Briefly around 1X10^6^ *L*.*major* promastigotes were cultured in 24 well plates and treated with 30μM of **C2NL**. The plates were incubated at 27°C for a time period of 48h. Promastigotes were examined using the 40X objective to study the effect of compounds on parasite morphology using EVOS FL Color Imaging System (Life technologies). To estimate the size reduction of the parasites, ImageJ v1.49 software was used. [25]

### SEM analysis of the treated promastigotes

Around 1X10^6^ *L*.*major* promastigotes were cultured in 24 well plates and treated with 30μM of **C2NL**. The plates were incubated at 27°C for a time period of 48h. Promastigotes were washed twice with PBS ph 7.2, adhered to poly L lysine- coated coverslips. Fixation was done in 2.5% glutaraldehyde solution (pH 7.4) for a period of 3 hours at 27°C. After fixation, the material was washed twice in PBS and dehydrated in acetone series, coated with platinum using Q150T Turbo- Pumped Sputter Coater. Cells were observed under a Nova NanoSEM field emission scanning electron microscope operating at 15 kV. Images were analyzed using the Gwyddion software. (http://gwyddion.net/)

### Mitochondrial potential reduction

To determine the effect of compound on induction of apoptosis and the associated reduction in mitochondrial potential, dual staining of *L*.*major* promastigotes was performed. Briefly 1X10^6^ *L*.*major* promastigotes were treated with 30μM of **C2NL** for a time period of 48h. Cells were harvested by centrifugation at 6010Xg and washed with PBS. Cells were incubated with Mitotracker Red CMXRos (Life Technologies) to a final concentration of 100 nM for 30 min. Cells were washed with PBS and resuspended in 100μl of 1X annexin binding buffer. Around 5 μL of Alexa Fluor^®^ 488 annexin V was added and cells were incubated in dark at room temperature for 15 min. After the incubation period, 400 μL of 1X annexin-binding buffer was added. Data acquisition was carried out using FACSCalibur (BD Biosciences) and analysis was done using CELLQUEST PRO software. 10,000 events from each sample were acquired. The representative plots are a result of three independent experiments.

### *Invivo* screening

The experiments were conducted according to the protocols approved by the Institutional Animal Ethical Committee of National Centre for Cell Science (NCCS, Registration number: 7/GO/ReBi/S/99/CPCSEA) under the Project number: EAF/2013/B-197. Mice were bred in the experimental animal facility at NCCS. BALB/c female mice, 8 weeks old with a body weight around 20–25 g were used in the study. *L*.*major* stationary promastigotes were cultured as per the procedure mentioned above. *L*.*major* promastigotes (2X10^6^ stationary phase promastigotes in 0.04 ml PBS) were injected subcutaneously in the left hind limb footpad of the mice. **C2** was dissolved initially in DMSO and diluted with PBS. The dose for *invivo* experiements was decided based on the standard dose of miltefosine used for treatment of visceral leishmaniasis (2.5mg/kg bwt). Keeping in mind the relatively higher IC_50_ value of **C2 and C3**, the compound dose was adjusted to 5mg/kg bwt. Oral administration of **C2** and **C3** (5mg/kg body weight) was done, treatment was started when the lesion thickness of 3mm was obtained. Mice were administered drug once daily for a period of 8 weeks. Mice treated with PBS were included as a vehicle control. Lesion size was monitored every week post infection using a vernier calliper. Lesion size has been expressed as footpad thickness (mm). Parasite load in the draining lymph nodes was estimated post euthanasia by limiting dilution assay for each of the treated and untreated groups as per the Taswell’s method. [26]

### Statistical analysis

A student paired t test has been used for data analysis. Analysis was done using SigmaStat software program (Binary Semantics, Pune, India). Results have been represented as Mean ±SEM (p<0.05 indicating statistical difference between the test and the control)

## Results

### In silico screening of the coumarin derivatives

**Fig 1:**
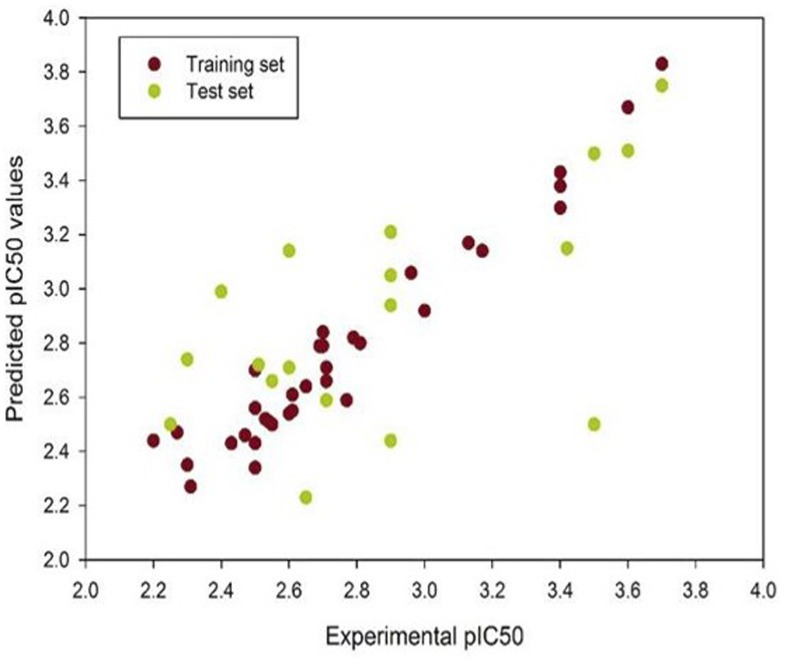
Screening of the compounds using FBQSAR. The scatter plot shows the predicted pIC50 values of the training and test set of the compounds used during the QSAR analysis. The top five compounds with an higher pIC50 were selected for further analysis.

**Fig 2:**
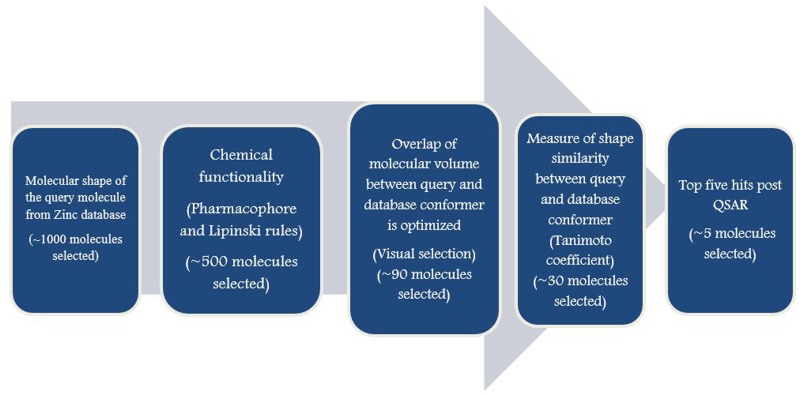
*Insilico* screening of the compounds. This figure depicts the procedure used during the screening of the compounds.

Coumarin derivatives obtained from the ZINC database were further screened based on their compliance with the Lipinski’s rule of five and Veber’s rules. Shape based screening method was used for screening of the derivatives and only those compounds with a Tanimoto combo score>0.6 were considered. (Table 1) Field based QSAR was performed to correlate the physicochemical properties of the compounds with their biological activities. The training set for the QSAR analysis was based on the standard coumarin compounds with known anti-fungal and anti-trypanosomal compounds. [12, 27, 28, 29, 30] The scatter plot showing the test and training set is represented in Fig 1. The model built was reliable as the q^2^ value of the model was 0.60 and the R^2^ value was 0.83. Based on the FB-QSAR results and the predicted pIC_50_ values, we selected the top five compounds for further *in-vitro* and *in-vivo* experimentation. The screening procedure has been outlined in Fig 2.

**Table 1:** Screening of the compounds using FBQSAR. Tanimoto combo scores of the top five compounds and their predicted pIC_50_ values.

S.No	Identifier	Tanimoto combo score	Predicted pIC_50_ value
1.	C1	0.65	3.70
2.	C2	0.84	3.61
3.	C3	0.75	3.46
4.	C4	0.85	3.45
5.	C5	0.74	3.41

### Anti-leishmanial properties of the compounds

The *in vitro* antileishmanial activity of **C1-C5** compounds was determined post 48h treatment of *L*.*major* promastigotes. The efficacy of the compounds was determined in terms of their IC_50_ values. Of the five compounds, compounds **(C1, C4 and C5)** showed little or no inhibition at all. (IC50 values > 1mM) while **C2** and **C3** showed anti-leishmanial properties with an IC_50_ value of 524 μM and 223 μM respectively. (Table 2) The percentage viability of the macrophages treated with **C2** was around 65%, while that of **C3** was around 85%. Thus there was no significant toxicity of macrophages seen with compounds **C2 and C3** at their IC_50_ values. In the *L*.*major* promastigotes treated with **C3** post 48h of treatment at the IC_50_ concentration, we did not observe any morphological changes while promastigotes treated with **C2** exhibited size reduction and loss of motility. Treated promastigotes were also noninfective to the macrophages (J774 cell line) and lost the ability to proliferate under *in vitro* conditions.

**Table 2:** IC_50_ prediction of the compounds in *L*.*major* promastigotes. IC_50_ values of compounds post 48h of treatment. Compounds giving less than 50% inhibition have been reported to have IC_50_ >1000 μM.

S.No	Identifier	Compound Name	IC_50_ value (μM)
1.	C1	*(3-(1,3-Benzodioxol-5-yl)-2oxo-2H-chromen-6-yl-acetate)*	>1000
2.	C2	*(6-Amino-3-(1,3-benzodioxol-5-yl)-2H-chromen-2-one)*	524
3.	C3	*3-(1,3-Benzodioxol-5-yl)-6-{[(1E)-2-furylmethylene]amino}-2H-chromen-2-one*	223
4.	C4	*3-(1,3-Benzodioxol-5-yl)-6-{[(1E)-1H-pyrrol-2-ylmethylene]amino}-2H-chromene-2-one*	>1000
5.	C5	*(3-(1,3-Benzodioxol-5-yl)-6-nitro-2H-chromen-2-one*	>1000

### Nanoliposome development of C2

**Fig 3:**
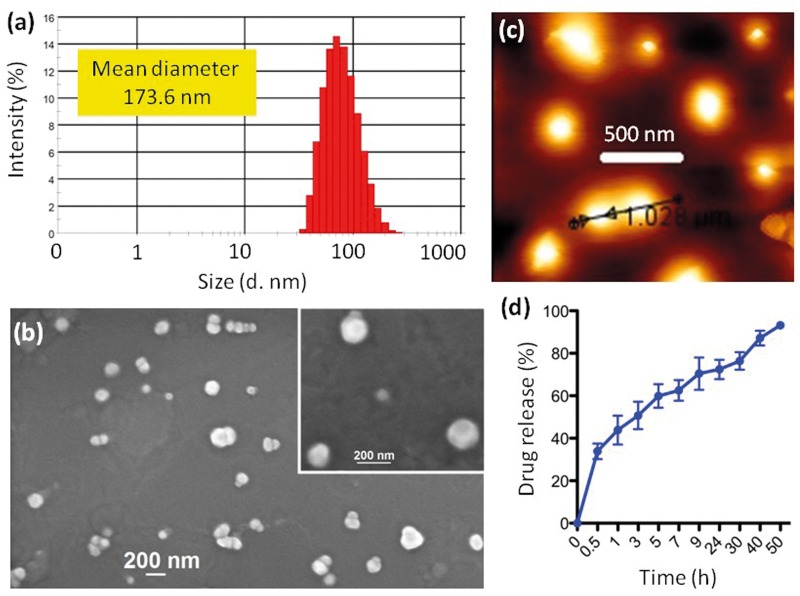
Characterisitics of the nanoliposomal formulation. a) Determination of the hydrodynamic diameter of the nanoparticles using DLS. Particles are sub 200nm in size b) Shape of the nanoparticles as observed by FESEM. Nanoparticles appear monodispersive in nature and are spherical in nature c) AFM characterization of the nanoparticles d) Release profile of compound 2 from the nanoliposomes. The nanoparticles ensure that around 93% drug release occurs over a period of 50h. The designed nanoparticles are capable of releasing the drug in a slow and sustained manner.

In order to improve the solubility and to study the anti-leishmanial properties of **C2**, the compound was encapsulated in the form of nanoliposomes. The mean drug loading was found to be 150 μg/ml (loading efficiency = 10%) by UV-Vis spectroscopy through a concentration versus absorbance calibration graph at λ_max_ = 256 nm characteristic of **C2**. The hydrodynamic diameter of the nanoliposomes was determined to be 173.6 nm through dynamic light scattering (DLS) (Fig 3a). Size, shape and morphology of the drug loaded nanoliposomes were determined by field-emission scanning electron microscopy (FESEM) and atomic force microscopy (AFM) (Fig 3b and 3c). From the FESEM and AFM images, it was clear that the drug loaded nanoliposomes were monodispersed and spherical in shape having sub 200 nm size. The nanoliposomal formulation developed was stable at both 4°C and at 37°C. From the release kinetics experiment (Fig 3d), it was quantified that almost 93.2% of the compound was released from the nanoliposomes over a period of 50h. Hence, the nanoliposome formulation of **C2 (C2NL)** ensured the slow and sustained release of the active compound over a long period of time.

### Efficacy of the nanoliposomes

**Fig 4:**
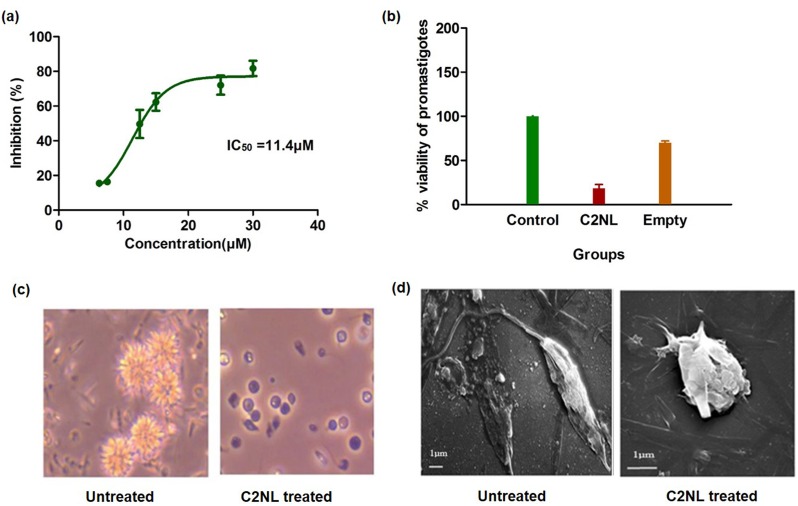
Testing the efficacy and cytotoxicity of the nanoliposomal formulation. a) IC_50_ prediction of C2NL. *L*.*major* promastigotes were treated for 48hours with 30μM of the C2 containing nanoliposomes. Bar graph indicates the percentage viability of parasites. A comparison has also been made with that of empty liposomes. Inhibitory effect was evaluated using MTT assay. b) Cytotoxicity of the nanoliposomes over J774 macrophage cell line. c) Microscopic analysis of the treated parasites. Parasites treated with C2NL show abnormal morphology and reduced motility. d) SEM images of the treated and untreated promastigotes. Treated promastigotes tend to round up and shrink in size. They also loose their flagella and become less motile. Bar = 1μm.

To determine the efficacy of **C2** nanoliposomes, we investigated the effects of **C2NL** on *L*.*major* promastigotes. Log-phase parasites were cultured in RPMI media and treated with **C2NL** with the concentrations ranging from 0–30μM. At the fixed points, cells were subjected to MTT assay to estimate the inhibitory potential of the nanoliposomes. It was observed that **C2NL** treated promastigotes show a dose dependent growth reduction with the IC_50_ value of **C2NL** ranging between 10–15μM. (Fig 4a) At the IC_50_ concentration, the viability of the macrophages treated with **C2NL** was around 80–85% indicating that the nanoliposomal formulation showed lesser toxicity to the host macrophages. (Fig 4b) In order to rule out the possibility of inhibition due to the liposomal mixture, we also performed inhibiton assay with empty liposomes. Percentage inhibition of empty liposomes equivalent to 30μM of **C2NL** over *L*.*major* promastigotes was around 31% further conforming that the inhibitory effect was due to the presence of **C2** rather than the liposomal mixture.

### Morphological changes

At a concentration of 30μM post 48h treatment of *L*.*major* promastigotes with **C2NL**, we also observed morphological changes in the parasites like reduction in size and motility (Fig 4c). To further confirm this observation, the percentage area reduction of the **C2NL** treated parasites was calculated which was around 48.1 ± 13% as compared to the untreated parasites. We also performed SEM analysis of the treated parasites which indicated that **C2NL** treated promastigotes appear to lose their flagella and exhibited size reduction in comparison to the control. This indicates that **C2NL** is capable of reducing the motility of the *L*.*major* promastigotes. (Fig 4d)

### C2 induces mitochondrial membrane potential reduction in *L*.*major* promastigotes

**Fig 5:**
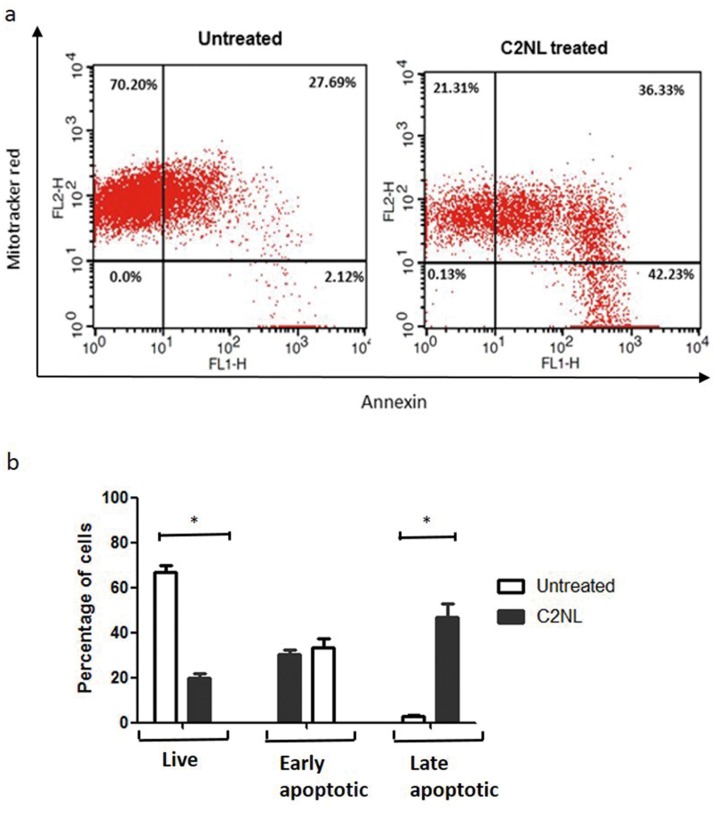
C2 induces mitochondrial mediated apoptosis in *L*.*major* promastigotes. a) FACS analysis showing the reduction of mitochondrial potential and the induction of apoptosis in the C2NL treated *L*.*major* promastigotes. Promastigotes were treated with 30μM of C2NL for a period of 48hours. The image shown is a representative image of three independent experiments b) Percentage of cells showing an increase in the number of cells in the late apoptotic phase in the treated samples as well reduction in the number of live cells. Experiment was performed in triplicates. Significance is indicated in the figures as *p<0.01. Error bars represent the standard deviation of the mean.

Mitochondrial membrane potential reduction is a characteristic feature observed in the cells undergoing programmed cell death.[31] **C2NL** treated *Leishmania* promastigotes were investigated for a change in the ΔΨ_m_. In addition, we also tried to check if **C2NL** induced apoptosis in Leishmania. Mitotracker dyes get aggregated within the normal mitochondria, which retain the ΔΨ_*m*_ giving a bright red flourescence. Externalization of phosphatidyl serine is one of the common feature of apoptotic cells. Cells undergoing apoptosis can be detected using Annexin V as it has the affinity to bind to the exposed phosphatidyl serine. [32] In the **C2NL** treated *L*.*major* promastigotes, the reduction in mitochondrial membrane potential and phosphatidyl serine externalization was studied by performing a dual staining with the mitochondrial potential specific Mitotracker Red CMXRos dye and the apoptosis specific Annexin dye. Cells were analyzed using flow cytometry and classified as early or late apoptotic based on the ability to retain ΔΨ_m_ coupled with the annexin exposure of cells. The *L*.*major* promastigotes were treated with 30μM of **C2NL** for a period of 48h before performing the staining. We observed a loss of the red fluoroscence signifying reduction of the ΔΨ_m_ in the **C2NL** treated promastigotes. (Fig 5a) In the untreated cells (promastigotes without treatment), there was a higher Mitrotracker red staining. In the treated promastigotes, a larger population of promastigotes had lower Mitotracker red staining indicating that one of the properties of **C2** is to lower mitochondrial membrane potential in *L*.*major* promastigotes. We also observed that the percentage of late apoptotic cells was higher in the treated sample. In the 48h treated sample, 36.3% of the cells were under early apoptoic stage while 42.3% of the cells were late apoptotic while in the untreated sample, 27.6% of the cells were under early apoptosis and only 2% of the cells were late apoptotic. This clearly indicates that **C2NL** was capable of inducing mitochondria mediated apoptosis in *Leishmania major* promastiogtes. (Fig 5b)

### Efficacy of C2 in *L*.*major* infected mice

**Fig 6:**
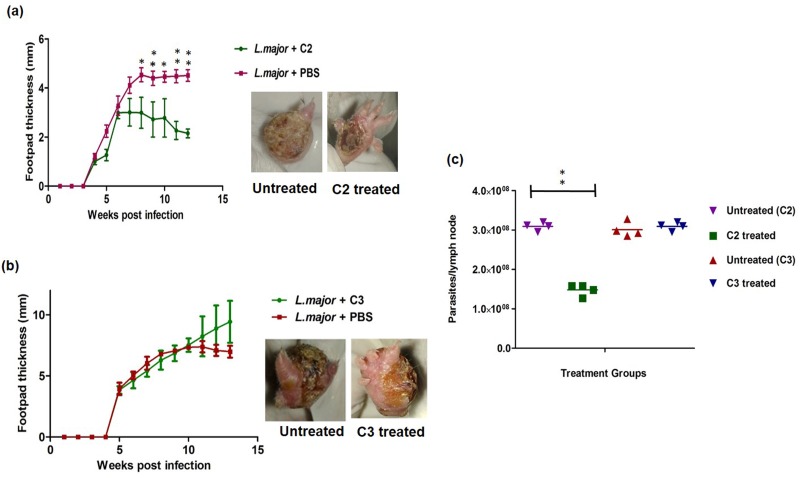
Course of *L*.*major* infection in mice treated with C2. a) Footpad swelling progression during drug treatment. Weekly recordings of footpad swelling are shown for the untreated and the mice treated with compound 2. Footpad swelling is expressed as the increase of the infected over the noninfected footpad. Data shown summarizes the (Mean±SEM) from 4 animals each. *p <0.05, **p<0.01. Photographs of the footpad at the end of the treatment indicate the progression of the ulceration. b) Footpad swelling progression of the miltefosine treated mice. c) Footpad swelling progression of the compound 3 treated mice d) Parasite load assay of the treated groups. Significance is indicated in the figure as **p<0.01. Every dot indicates one mice.

Based on the in-vitro experiment the effect of **C2** and **C3** was tested in BALB/c mice infected with *L*.*major*. Oral administration of the compounds at a dose of 5mg/kg/bwt orally was done for a period of 8 weeks. The progression of the infection was assessed based on the footpad thickness. The footpad thickness of the mice treated with **C2** was significantly lower as compared to the untreated mice. (Fig 6a) No reduction in footpad thickness was seen in **C3** treated mice and it was seen that this compound was ineffective (Fig 6b). The parasite loads in the draining lymph nodes of the mice treated with **C2** was lower as compared to the untreated mice (Fig 6c).

## Discussion

Coumarins are potent anti-fungal compounds and a few studies have also reported that coumarins have anti-leishmanial properties. Though their exact mechanism of action has not yet been deciphered, their ability to enhance the phagocytotic activity of macrophages is well known. [33] We identified a set of coumarin derivatives and tested their efficacy on *Leishmania major*. Shape based matching of coumarin derivatives with their parent compound was done, which excluded the compounds having lower similarity. 3D-QSAR which exploits the 3D properties of the ligands to predict their biological activities was used for further screening. We further tested these compounds over *L*.*major* promastigotes to identify the compounds with anti-leishmanial properties. It was seen that treatment with compound **2** leads to a dose-dependent inhibition of proliferation. To further improve the solubility of the compound and to explore the anti-leishmanial properties of this compound, nanoliposomal formulation was prepared. Parasites treated with **C2** showed drastic reduction in motility and reduction in size. They also lose their ability to infect the macrophages (J774 cell line). Dual staining with annexin-mitotracker red indicated that the nanoliposomes developed were able to initiate mitochondrial mediated apoptosis in the treated parasites. Based on the *in vitro* screening, we tested the efficacy of **C2** and **C3** in *L*.*major* infected BALB/c mice at a dose of 5mg/kg/bwt. Our observations indicate that there was a healing effect in the footpads of mice treated with **C2**. Together, our study indicates that **C2** serves as an intial hit which could be further modified and developed for realizing its potential as a potent anti-leishmanial compound.

## Conclusion

There is an urgent need to design and develop novel anti-leishmanial compounds due to various problems associated with the current chemotherapeutic. We have designed coumarin derivatives using *in silico* approaches. Five of the inhibitors were selected for further *in vitro* experiments, their cytotoxicity and efficacy against *L*.*major* has been tested. Parasites treated with compound 2 and its nanoliposomes showed significant morphological changes. Thus the possibility of using coumarin derviatives could thus be exploited towards treatment of cutaneous leishmaniasis.

## Supporting Information

S1 Fig Schema for the synthesis of C2. (TIF)

S2 Fig NMR Spectra of C2. (TIF)

S3 Fig IR Spectra of C2. (TIF)
