# Supplementary figures and images for: Biological Activity of Coumarin Derivatives as Anti-Leishmanial Agents
Source: PLoS One. 2016 Oct 21;11(10):e0164585. doi: 10.1371/journal.pone.0164585 (PMC5074534; doi:10.1371/journal.pone.0164585)

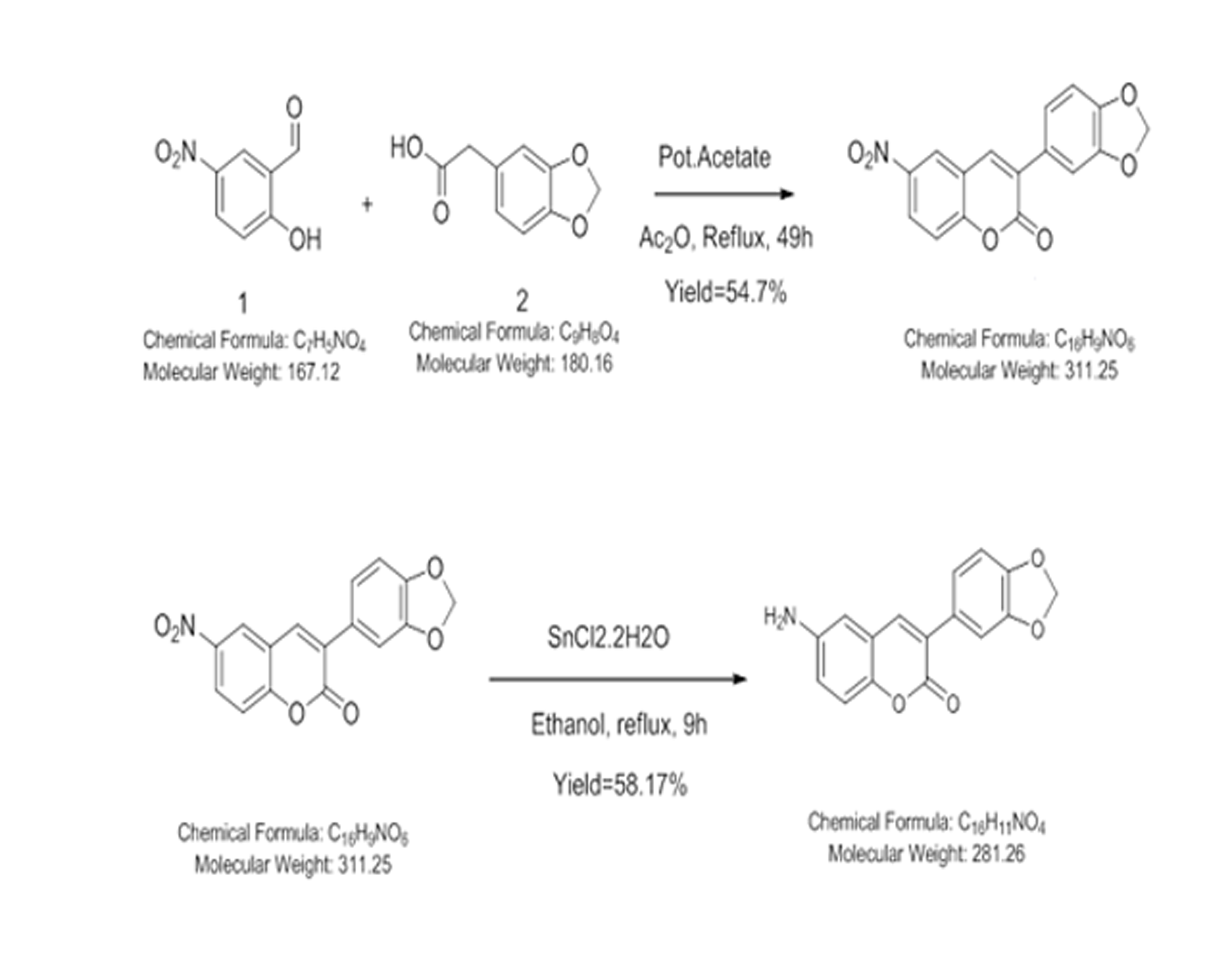

Supplement: S1 Fig — (TIF) [file pone.0164585.s001.tif]

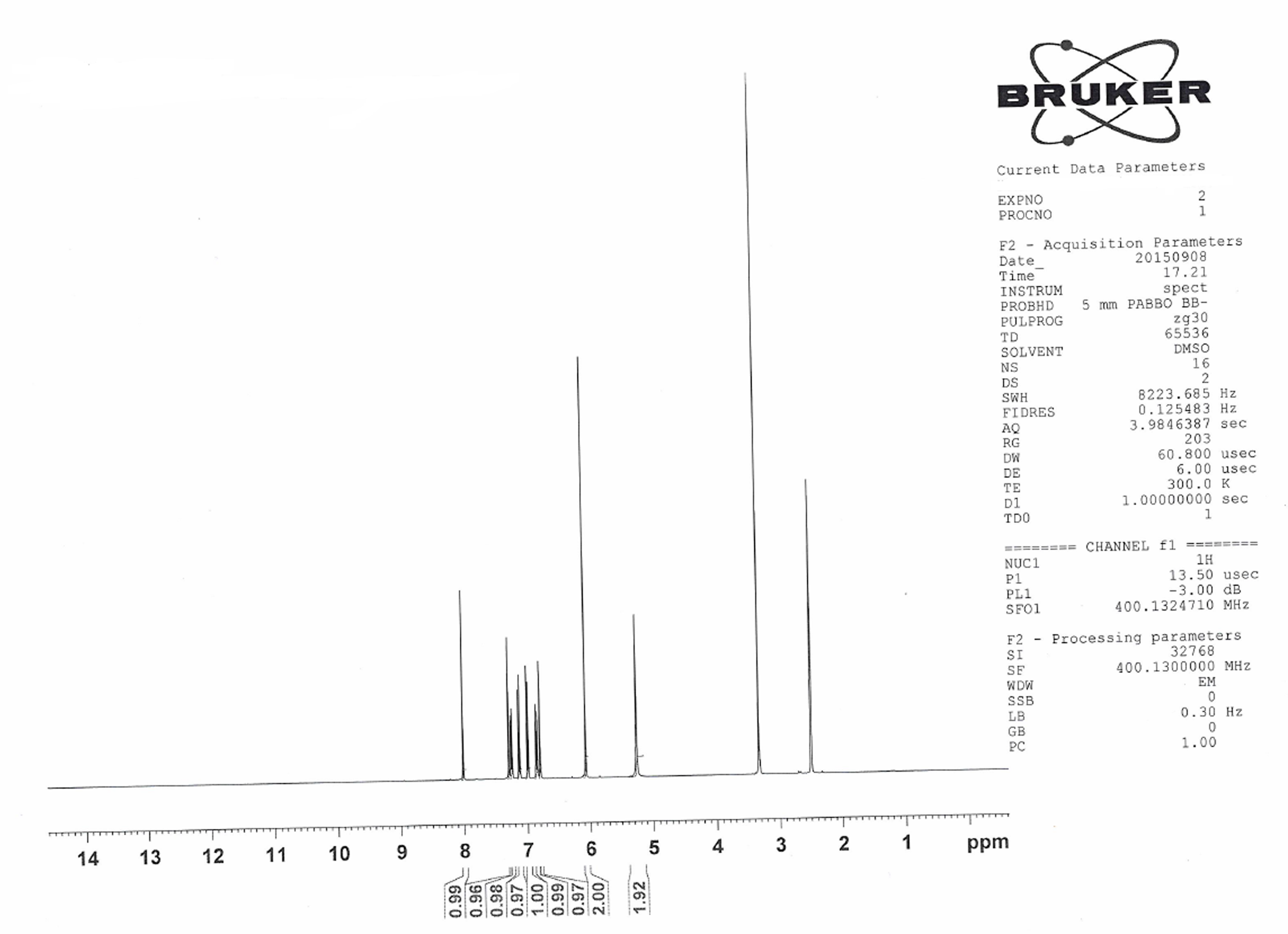

Supplement: S2 Fig — (TIF) [file pone.0164585.s002.tif]

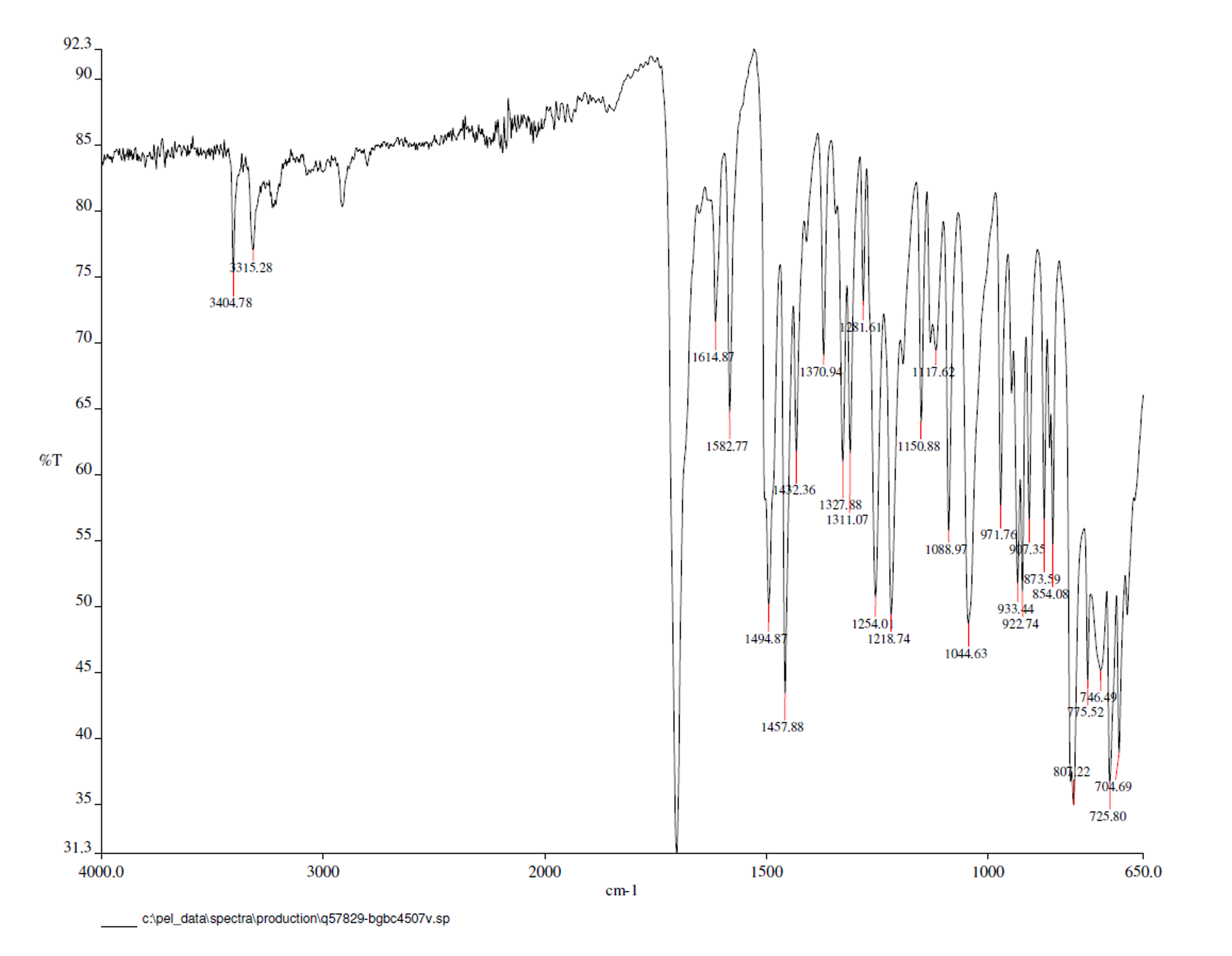

Supplement: S3 Fig — (TIF) [file pone.0164585.s003.tif]
